# Being-in-the-Chemotherapy-Suite versus Being-in-the-Oncology-Ward: An Analytical View of Two Hospital Sites Occupied by People Experiencing Cancer †

**DOI:** 10.3390/cancers9060064

**Published:** 2017-06-05

**Authors:** Catherine Hughes, Kate van Heugten, Sally Keeling, Francisc Szekely

**Affiliations:** 1 Department of Social Practice, Unitec Institute of Technology, Auckland 1025, New Zealand; 2 Department of Human Services and Social Work, University of Canterbury, Christchurch 8140, New Zealand; kate.vanheugten@canterbury.ac.nz; 3 Department of Medicine, University of Otago, Christchurch 8011, New Zealand; sally.keeling@otago.ac.nz; 4 Research and Enterprise Office, Unitec Institute of Technology, Auckland 1025, New Zealand

**Keywords:** palliative care, chemotherapy, oncology, ethnography, end-of-life care, place

## Abstract

How do people with cancer occupy places within the health system during their journey through palliative care? The answer to this question was explored by the authors as part of a wider ethnographic study of eight people’s journeys from referral to palliative care services to the end of life. This article reports on findings that have emerged from ongoing analysis that has been completed in the years proceeding data collection. An ethnographic research design was used to collect data about the participants and their family members over a three-year period. Data was collected using participant observation and semi-structured interviews. Over 380 transcripts based on field note entries and taped interviews were produced during the 1121 h of contact with participants and family members that made up the research period. Analysis of these texts identified two focal sites within Christchurch Hospital that were occupied by the participants. These were the Chemotherapy Suite and the Oncology Ward. Drawing on literature concerning previous anthropological analysis, research was conducted to understand how places affect people and how people affect places. The researchers have used a model outlined by the American ethnographer Miles Richardson to analyse two distinct sites within one hospital. As explained in Richardson’s article, whose title is used to model the title of this article, a sense of place becomes apparent when comparing and contrasting two sites within the same location. Richardson’s article is highly interpretative and relies not only on pre-existing theoretical frameworks but also on personal interpretation. The same approach has been used in the current article. Here, ethnographic methods require the researcher’s interpretation of how participants occupied these sites. Following this approach, the Chemotherapy Suite is presented as a place where medicine dominates illness, and appears as distinct from the Oncology Ward, where disease predominates and death is secreted away.

## 1. Introduction

The New Zealand Palliative Care Strategy [1] defines palliative care as follows:

Palliative care is the total care of people who are dying from active, progressive diseases or other conditions when curative or disease-modifying treatment has come to an end. Palliative care services are generally provided by a multidisciplinary team that works with the person who is dying and their family/whanau

(2001, p. 2).

Palliative care has developed as an area of specialisation, providing an alternative model of care for patients with incurable illness [2]. Despite the negative connotations of death and dying commonly associated with the term “palliative care”, it has been proposed that educating people about palliative care may be a more useful strategy than simply changing its name [3]. The hospice model is widely accepted as an ideal framework within which to provide palliative care [4]. The model has been established in a variety of places and settings, including hospitals, aged care homes, day care centres [5,6], and residential [7,8]. Fordham and Dowrick [9] researched these types of sites and indicated that people who had access to specialist palliative care teams received a slightly higher quality of care. They identified the need for research which considers the general care of the dying instead of focusing on specific hospital or hospice populations. Additionally, they noted the need for research to be carried out with the people who are dying as opposed to their carers.

Despite the proliferation of articles related to aspects of palliative care, researchers have continued to highlight the need for empirical studies that focus on “people experiencing dying”. Wright et al. [10] have addressed this question in a study of eight oncology clinics across the United States and found that patients who received palliative chemotherapy within four months of dying were more likely to seek intensive medical care and therefore more likely to die in hospital than those who did not. Patients receiving chemotherapy tended to be referred to hospice late and they were less likely to die in their preferred place of death.

In a recent study of 36 nations, findings indicated that people in Aotearoa New Zealand (ANZ) are as likely to die in hospital 34% as in residential aged care (RAC) facilities (31%) or at home (35%). In comparison to the other countries surveyed, ANZ had one of the lowest rates of hospital deaths and one of the highest in RAC facilities. The researchers suggest that the reason for the high number of death in RAC facilities may be indicative of the number of hospital discharges of older people into RAC at the end of life [11].

Ever since Copps [12], researchers have continued to note the lack of empirical research that considers patients’ and their caregivers’ perceptions of palliative care [3]. Furthermore, research focused on places occupied by palliative patients is often concerned solely with the place of death [13,14], and fails to recognise the importance of the places these patients inhabit on their journey to the end-of-life, which is where the current study situates its particular interest.

Although no literature concerned with palliative patients’ occupation of sites within the hospital was identified, there has been some research on the impact of palliative chemotherapy on patients. However, Wright’s research focuses specifically on the relationship between chemotherapy and eventual place of death, and its scope is, therefore, limited [10].

Radley and Taylor [15] produced a highly relevant ethnographic study, which needs to be mentioned here, even though it is not centred on *palliative* patients but on the experiences of patients in hospital wards. Their article addresses the embeddedness of patient’s experiences by focusing on patients’ reliance on the ward and the objects within it for their physical existence. They also considered the physical sensations that accompanied many of the medical interventions required. As a result of this dual focus, the participants in the ward were firmly embodied.

Hospital technology and institutional settings embody the ideologies of modern medicine in which the promise of cure is premised on the requirement of cooperation. To engage that ideology in hospital is not just to sign up to a set of beliefs but, in one’s own turn, to embody them in acts of acquiescence and resistance at a corporeal level. This bodily endorsement of the object’s constraint or intrusion is the anticipation of its purpose. Taken as a totalizing exercise, this is the act of becoming and remaining a hospital patient

([16], pp. 93–94).

This quote makes apparent the relationship between places and people, which is also the focus of the current article.

With the intention of exploring how people engage with the health system and develop meaning from referral to palliative care, the first author of the present article carried out an in-depth ethnographic study as a doctoral thesis supervised by the second and third authors [17]. The study followed eight people with incurable cancer, and members of their families, through the health system from the point of referral to palliative care, to the end-of-life. Whilst the ethnographic study was a wider exploration of participants’ journey through palliative care, the location of people in particular places and spaces produced a significant body of data, which opened the thesis to new interpretations which are discussed in this article.

## 2. Methods

Ethnography draws on people’s ability to learn a new culture by being immersed in particular environments, thereby becoming part of the culture that is being studied [18]. O’Reilly describes ethnographic methodology as “a methodology that acknowledges the complexity of human experience and the need to research it by close and sustained observation of human behaviour” ([19], foreword, n.p.). The researcher may be an observer in some situations and a participant in others. The ultimate goal is that, through natural engagement in people’s lives, the researcher’s presence will cause little disruption and eventually the participants will forget the researcher’s presence and act in a natural manner. This gives the ethnographer a semi-overt role, in that the participants know he/she is there as researcher but they become less conscious of this explicit role. A more in-depth discussion of the role of the researcher as participant and observer was outlined by Hughes, van Heugten and Keeling [20]. One of the goals of ethnographic enquiry is for the researcher to move from being what Okely [21] called the “outsider” to being the “insider” in order to engage in the participant’s world in a natural manner. Because of this the researcher makes no claims about neutrality, openly acknowledges the reciprocal relationship with the participants, and draws on the knowledge of the researcher “self” [22] in the ethnographic text. The researcher was a mature qualified social worker undertaking doctoral research. The second and third authors provided expertise from ethnography and social work.

### 2.1. Ethics

Initial meetings with participants took place in their homes, and in most cases the first meeting involved other family members. At this first meeting the research was explained and informed consent was gained from those who were present. Ongoing consent to collect data was gained from new family members and friends when they were introduced to the researcher. In a similar manner, the researcher spoke to health professionals about the project and sought consent from them to be present and to take notes while observing interactions between the consented participants and health professionals.

Given the sensitive nature of the topic and the involvement of vulnerable participants, ethics approval was sought and granted by three relevant bodies: the provincial Canterbury District Health Board’s “Canterbury Ethics Committee”; the Regional Palliative Service’s “Nurse Maude Ethics Committee”; and the University of Canterbury’s “Human Ethics Committee”.

### 2.2. Participants

To be included in the research, participants had to be over 18 years of age and speakers of English, because being able to understand the participant’s conversation is vital to ethnographic research. The participants had to have been referred to a local palliative care team. In Aotearoa New Zealand, a prognosis of less than 12 months’ life expectancy is a precondition for referral to palliative care services [23]. Although there was no minimum time requirement for the participants’ involvement in the study, the researcher decided to exclude data from participants who had been visited by the researcher less than three times. Data collected in those cases would have given the researcher an incomplete picture of participant’s experiences, and would have made generalisations impossible. The small number of visits was due to the fact that these potential participants died during the research and earlier than predicted. This resulted in eight participants being included in the study. They were recruited with the assistance of community-based hospice nurses and the hospital palliative care team. The researcher observed and participated in the journeys of these eight people with a diagnosis of incurable cancer, from diagnosis to the end-of-life. It is important to point out here that the researcher was with all the participants in the hours prior to their death, so the concept of reaching the end of life was always experienced directly.

Other participants included in this ethnographic research were family members, hospital staff, volunteers, other patients, and visitors to the hospital. Of the eight patients recruited as participants, four were men and four women. Seven of the eight participants lived with a partner, while the remaining participant lived with her daughter. The participants’ ethnic backgrounds varied but included New Zealand Māori, Pakeha (NZ European), Hungarian, French, Scottish and Irish. Patients were aged in the range between mid-thirties and late-seventies, with only two participants being of retirement age (over 65). There was also variation in the socio-economic status of participants, from some who identified as struggling financially throughout the journey, to others who classed themselves as financially comfortable. It would be fair to say, however, that each of the participants’ financial status was negatively affected by their illness, as all but one were employed at the time of diagnosis.

### 2.3. Location

Christchurch Hospital is located near Christchurch City Centre, New Zealand, and is bordered by the Avon river and 21 hectare botanical gardens on one side [24], and the 165-hectare Hagley Park on the other [25]. Christchurch Hospital is one of the four largest teaching hospitals in New Zealand [26]. It has one of the busiest emergency departments in Australasia, seeing over 83,000 patients a year. There are also over 40,000 inpatients and 300,000 outpatients who receive services from the hospital each year. The hospital has 500–550 beds for inpatients [27]. The research outlined in the present article was completed prior to the 2010–2011 Canterbury earthquakes. Since the 2010 earthquake series there have been a few disruptions to the location and services of the Canterbury District Health Board. However, the location of the Oncology Department, the Chemotherapy suite, and the Oncology Ward have remained the same. Christchurch is the third largest city in New Zealand, with a population of 341,461, which represents a drop from 348,459 at the time of the previous census in 2006 [28].

The two sites that are the focus of this research are the Chemotherapy Suite and the Oncology Ward, both located within the public hospital buildings.

### 2.4. Ethnographic Data Collection

The researcher accompanied the eight participants and their family members on the majority of their visits to the Chemotherapy Suite at Christchurch Hospital and spent 123.6 h with them in the Oncology Ward during admissions to hospital. Data relevant to the theme of “occupation” of these sites was logged by using jotted field notes, which formed a selective representation of each field-visit. The field notes were then transcribed each evening over the period of the fieldwork, as suggested by Lofland and Lofland [29]. Emmerson et al. [30] have shown that this is one of the primary methods used to collect data in ethnographic research. During this time, there were a total of 380 transcripts produced, which made up the entirety of the ethnographic record of the researcher’s observations. The transcripts included taped/transcribed interviews as well as observational field notes. The following table (Table 1) shows the amount of time spent with each of the eight participants (in minutes) and how this varied depending on the life span of the participant. The table also indicates type of contact made, or place of contact. The amount of contact is totalled in the sum-total column and then the amount of time writing up the field notes is added to give an overall data collection time in minutes. Minutes are then calculated as days, weeks and months in Table 2.

To further elaborate, Table 1 provides accurate details of the time spent (in minutes) with each participant in the various settings or modalities that were encountered during the research. Table 2 indicates the total contact time with the eight participants and their family members, including phone time, face to face contact and travel time (with or without participants), which is shown to be the equivalent of eight hours a day, five days a week, for seven months. The amount of time it took to transcribe the jotted field notes into typed transcripts is equivalent to eight hours a day, five days a week, for over seven and a half months. The process of collecting and collating the data required for the study was, therefore, equivalent to eight hours a day, five days a week, for over fourteen and a half months. The two areas of focus in the current article are the Chemotherapy Suite and the Oncology Ward. Table 2 indicates that the researcher spent eight hours a day, five days a week, for over 2 weeks in the Chemotherapy Suite and eight hours a day, five days a week, for over 3 weeks in the Oncology Ward.

**Figure 1:**
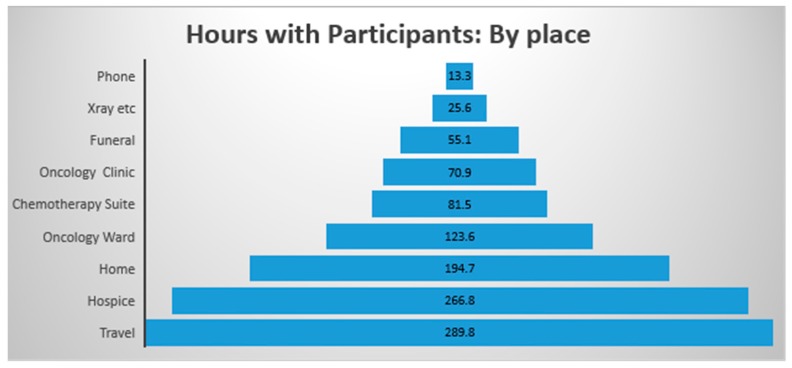
Amount of time spent with participants and their family networks.

The chart below (Figure 1) shows the time in each place by hours. Travel was to participant’s homes and hospital, as well as picking participants up and taking them to appointments and home again. Some travel included face to face time with participants. Further breakdown of traveling time was not recorded.

Figures relating to the places of interest in the current article, the Chemotherapy Suite and the Oncology Ward, indicate that the researcher spent 81.5 h in the Chemotherapy Suite and 123.6 h in the Oncology Ward. Quantitative data has been recorded in relation to the research methodology; including the amount of time spent in contact with the participants and their families, as well as the amount of time required for the transcribing of field notes. However, it was not the intention to provide quantitative data for the purpose of data analysis, but purely for consideration of the commitment required to complete or replicate an ethnographic study of this nature.

The intention was to record in-depth qualitative data; therefore, no attempt was made at quantifying the “data” but rather providing samples of what the participants said or the researcher observed. The choice of not quantifying this data is because some of the field notes are based on the subjective experience of the researcher as opposed to verbatim quotes of conversations. This is a similar approach to that used by Richardson [31], who spent time on the two sites that he compared, taking subjectively-filtered notes of things he observed. Richardson explains;

In actuality, from a purely ethnological, neutral-observer point of view, the behaviour occurring in the settings varies over a wide range and the recording of that variation… is extremely difficult. A portion of what people do and say may not be critical to either incorporating or challenging the emerging definition

(p. 426).

This quote explains why data quantification is not necessary in a viewer-centred approach and why reliance on the researcher’s observations are considered valid in an ethnographic study. For example, in the current study, if one of the participants was talking to another patient in the chemotherapy suite, the researcher would note that they were interacting and what the interaction was about, as well as the tone of the conversation, as opposed to the words being said. In some cases, however, if the content of the conversation was relevant to the research, partial conversations were noted verbatim.

The field-notes contained reference to the age, colour, and style of furnishings, participants’ reactions or thoughts about new places they were encountering, and how they behaved in familiar settings. Particular attention was paid to the size of each space, the proximal distance between people, where personal boundaries lay, and how this differed in each setting. The sites occupied by the participants could not be reduced to their material settings but included ways in which these settings enabled abstract relationships and interactions between participants and their environment as well as between participants and other people.

The initial intention of the research [17] was to fathom the participants’ abilities to understand and make meaning from referral to palliative care. However, as the analysis of the ethnographic texts collected during initial research began to deepen, it became increasingly noticeable that the data often spoke about places occupied by the participants. This theme turned out to be too important to be left unattended. Hence, the need to write the present article based on the interpretation of place-relationships emerged from this body of data.

## 3. Findings and Theoretical Framework

The findings will be presented as interwoven with the two-primary theoretical/methodological frameworks that provide the structure and analysis of the findings. It is somewhat contested in ethnographic analysis to separate the findings from the discussion as integration, interpretation and ongoing contrast and comparisons with existing research is regarded as an ever-deepening circular iterative process. Whereas the findings section introduces the theories, the subsequent section, discussion, is best conceived as the application of the two theories that have influenced the interpretation of the data. van Gennep’s theory draws on the individual journey metaphor and Richardson’s theory is more summative in that it goes back to the material level and draws on the total body of observational data—the participants’ journeys, plus this material set of observations.

### 3.1. Transition and Rite of Passage—Van Gennep’s Theory

It was noted by the researchers in the current study that, after diagnosis, many of the participants inhabited spaces within their communities less often than they did prior to illness. The process involved in this transition has been referred to as a rite of passage; a journey within which the people experiencing illness are relocated by the nature of their illness from one social category to another [30]. Arnold van Gennep [32] was the first ethnographer to classify rites of passage. He distinguished between rites of separation, rites of transition, and rites of incorporation.

Separation, which is the aspect that the present study is most concerned with, and all the actions and rituals associated with it, is marked by detachment from familiar activities and places previously occupied, such as places of employment, and by the consequent development of a new identity. Insofar as the present study is concerned, the seven participants who were employed at the time of diagnosis either left employment or significantly reduced the amount of time spent at work. Their admission to hospital was a culmination of this stage of separation and, as such, hospitalisation was understood as a partial transmission of their agency to clinical teams and ultimately to palliative-care personnel. This was problematic for participants who previously believed that they had full control of their lives. This realisation added anxiety to their overall experience. The eighth participant, who was retired, moved from her home in a small rural town to the city, to live with her daughter. The fact that all participants changed their immediate “everyday” environment shows that palliative-care patients experience a significant change in their attitude towards familiar places. These changes may not have been consciously regarded by participants as “rites of passage”. This concept was extracted from ethnographic literature and applied by the researchers to interpret the information provided by the participants. A decision was therefore made to incorporate van Gennep’s theory as it provides a useful lens through which to view the changes that occurred in their lives. The rites of transition they performed after the initial separation shifted their thinking significantly. Instead of focusing on what they might gain from living, many of the participants began to think of what they would lose from dying, and how the process of dying, of illness, would change their lives. The separation theorised by van Gennep is, in their cases, not only an estrangement from familiar places but also a shift in their understanding of the self. On a visit to Helen (Participant 5; for ethical and privacy reasons names used in this article are pseudonyms) at her home, she made the following statement:

“It was the bone cancer that threw me because it meant a whole change of lifestyle. We used to go away skiing all the time and I knew that I wouldn’t be able to do that anymore. I also used to work in the garden a lot and used to be able to haul huge wheelbarrows of mulch and stuff. Now suddenly I couldn’t do any of those things. I had to change my life”.

[33]

Helen’s statement shows how separation from familiar settings affected the participants, insofar as she perceived this separation as detrimental. Numerous other examples provide a sense of isolation and marginalisation felt by the participants. The following examples are also drawn from the researcher’s notes on Helen:

Helen explained that she had been living with cancer for almost three years and mentioned that she no longer attended the cancer support group that she used to go to regularly. She said that she just kept in touch by phone with a few of the women who were still alive. When asked by the researcher why she had stopped going to the group, she explained that as time passed she went to the group less and less as it became an emotional struggle for her to hold onto hope that she might live, when so many of the women in the group died of their disease

[33].

Helen also talks about a cross-stitch group she belonged to. They have lunch, a natter, and then sew from 12.30 to 3 p.m. Helen used to go there during chemo and they all knew she had cancer but no one ever asked her about it. ‘They didn’t even ask about the tumour in my head.’ Helen stopped going every week and attended fortnightly instead. Then she went less and less, as she couldn’t stand their lack of acknowledgement of her illness

[33].

This initial reduction in social spaces, related primarily to employment and social activity, was later offset by engagement with health services, which resulted in the participants’ spending more time in new spaces of social significance, namely the hospital oncology department, the oncology ward, and the local hospice.

As already shown, van Gennep’s [32] rites-of-passage theoretical framework can be applied to people living with loss, more specifically with cancer, as is the case of the present study, or to those living with the knowledge of impending death, as all of the above mark their presence in a transition stage. In van Gennep’s work this is referred to as a temporary state of in-betweenness, marked by detachment from life, a sense of isolation, and marginalisation: a state of liminality. Froggatt [34], who sees hospice as a clear illustration of van Gennep’s *limen,* states that one characteristic of this special state is “a concern with a new and different status” ([34], p. 128).

The following extracts from the field notes refer to two different stages of Jack’s journey (Participant 1) and provide examples of how he experienced the transition of status from being employed and independent to becoming part of the hospital setting. The following field note was made during a home visit to Jack.

We walked into the dining room and took our usual seats at the table. As Jack sat down, he said, “my memory is getting so bad, I have an appointment at work at 1pm and I need to hang the washing out first”… I (researcher) asked him what the work appointment was about, was it a farewell, and he said, “You might be right. They have been holding my job open for me, but I don’t think they can do it anymore. I could never go back now, anyway”

[33].

Jack’s last comment was one of very few acknowledgments that his illness was progressing and that the likelihood of him ever getting well again was very slim. Up until this point, Jack’s language was often about fighting the illness and getting well.

The second extract below was from one of Jack’s visits to the Oncology Clinic, which adjoins the Chemotherapy Suite. One of the clinic rooms can be accessed from the clinic hallway or via the Chemotherapy Suite. Its purpose is therefore twofold: it can be used for patients who may be quite sick during chemotherapy or for patients visiting the Oncology Clinic. This visit today has been arranged as Jack has been in a significant amount of pain all weekend and his oncologist thought he should come into the Oncology Clinic with a view to being admitted to the Oncology Ward. Driving into the hospital Jack explained that he had been in a lot of pain and was very disappointed about having to go into hospital. We arrive at the hospital and go into the Oncology Clinic where Jack is shown to a clinic room so he can lie down on a bed.

After a while, a nurse came in and got a box of gowns out, and then another nurse came in and got some chairs. Jack didn’t mind, though, because the room was familiar to him. He said, “this used to be my chemotherapy room”. He sounded a bit wistful, as chemotherapy is now a thing of the past for him. You could almost hear the lost hope in his voice

[33].

When Jack commented that this used to be his chemotherapy room, he smiled sadly in acknowledgment that this time had now passed. He is upset about having to be admitted to the Oncology Ward today as he sees it as acknowledgment of the progression of his illness. Jack´s wife also made the comment during a recent home visit that she does not think it will be long now until he goes into the ward to stay. There is an implication that admission to the Oncology Ward is for end-of-life care.

Being moved from the Chemotherapy Suite to the Oncology Ward is a process of transition between two places but also, more importantly, from one phase of the illness to another. Moreover, the Ward is a place where people often die: the last stage in a journey that started with diagnosis. Rites of passage taking place between these stages include separation from the old place and transition from the old setting to the new one, all of this unfolding under the umbrella of institutionalised care, with its own rituals and ceremonial patterns (admissions, discharges, and so on).

Each of the participants understood this transition in their own way. However, every participant who was undertaking treatment acknowledged sadness about the end of treatment and expressed their concerns about what would happen next. During his last visit to the Chemotherapy Suite, Daniel (Participant 2) made the following comments:

“It’s strange, but I like coming here and I don’t find it morbid, because all the nurses are so nice and the people here are all going through the same thing as me. I will really miss this place and seeing the other patients”.

The nurse had come over to speak to Daniel while I was out getting him a cup of tea. When I came back and sat down he said, “The nurse said that’s it. No more work for Daniel. She said you’re on your way out. She said your days are over”. Daniel added, “I’m still going to keep pushing, don’t you worry. I don’t want to go yet”. I asked Daniel how he felt about her saying that. Daniel said he needed to know. He said he will slow down and still keep pushing for that goal

[33].

Another example of the realisation that existence was impermanent can be seen in the following field note extract from a home visit to Alice (Participant 4).

Alice has made the decision to stop treatment because the side effects are making her so ill. She said that she didn’t think that she realized before that she was actually going to die because she was so used to being sick, so used to having cancer, and even though she knew she would never survive the cancer, she never really accepted the fact that she was going to die from it. She feels now that there is no future. That there is no hope. She thinks it will be fairly soon

[33].

This realisation has a significant impact on the participants. The researcher noted that after this time there was often a further reduction in the number of places that participants occupied and the number of people they engaged with. Passage became a matter of identification with the place of dying, to the point where the participants were reduced to the smallest place possible: the room where they were going to die. This could be either a bedroom in their own homes or, for those who chose hospitalisation, their respective single-bed rooms in the Oncology Ward. An example of the reduction of space which translates into a reduction in relationships is provided by the field notes of a home visit to Alice, when she was explaining that she did not want to spend time with anyone but her husband, Dennis, now.

Alice said that Dennis’s friends think that she is insecure and neurotic but they don’t understand that if Dennis leaves in the morning and things are not okay, that could be the last time they see each other and it would be horrible if she died on the day they had had an argument

[33].

Relationship is crucial here. As pointed out by Richardson [31], a socially-significant place is defined not only by its position and situation but also by the relationships it engenders. As the latter become more prominent, the former appears to fade. Alice had very little energy to engage with people, so her focus was on her relationship with Dennis. She didn’t want to see friends or go out to any social events. For her, the only relationships that really mattered were with Dennis and her sons. During this part of Alice’s illness, she began to speak more and more about what would happen to Dennis and her sons once she died. She spoke about wanting Dennis to carry on with his life, and to meet someone else to share his life with. She also spoke about what she saw as the appropriate timeframe in which this could happen, and which would feel okay for her. She thought that if he started going out with someone else within a year of her dying, she would be pretty upset about that.

It is interesting to note that participants such as Alice had ideas about the length of processes that led to the final phase of passage, reincorporation; which is more relevant to family members’ life after bereavement, than it is to the participants. This sentiment was also expressed by Helen during one of the researcher’s last home visits to her:

She said that she has tried to encourage Chris [her husband] to take up golf and play more often. She said: “he needs to get into the habit of doing things on his own because I’m not going to be around for much longer”. She said that although she sees friends on her own, Chris doesn’t really, and she worries about how he will get on when she is gone. She said that she knew he would be very lonely. She also said that he would meet someone else one day. I asked her how she felt about that. She said that he was young enough and she hoped that he would find someone else to be happy with. She said that she hoped he would wait at least a year, though

[33].

Van Gennep [32] explains that rites of reincorporation entail the formal enactment of funerals, or memorials. This stage involves family members letting go, taking time to grieve, and gradually re-joining society. An example of how the process of grieving together as a family can assist in the transition that occurs in this last stage is provided in the following extract from Tom’s (Participant 8) field notes on the day the researcher attended his funeral:

Jenni (Tom’s, sister) says that they have all been hanging out with each other since Tom died and it has been really nice. I asked her if they took him home and she said that they had taken him back to his place and he was in a room just off the lounge. She said it was a really nice feeling to have him there. I asked her how her sister Sara coped with it because she had made the comment at the hospice that she wouldn’t be going near him when he was dead. Jennie said that Sara had been great too and asked me if I knew that Sara had been there when he died, and I said no. She said, yeah Sara said that he just took one final breath and sighed and then he was gone. She said it was really nice and peaceful

[30].

As shown so far, van Gennep’s [32] triadic model of rites of passage is readily applicable to the study here described. The Chemotherapy Suite and the Oncology Ward of Christchurch Hospital were unfamiliar places, where the participants were brought only to fulfil medical requirements for care. Therefore, they perceived and represented the new settings in terms of ruptures, or separations from their familiar places and, consequently, from the world at large. Added to this is, of course, the idea of being on the threshold between life and death, an idea that occurs as new, or unexpected, to the participants.

What is significant in relation to the two sites observed by the researcher, is that the very architectural layout of the building in which these places are situated supports the notion of passage, of transition. To move from the Chemotherapy Suite to the Oncology Ward, patients had to literally journey across the building. Situated at opposite ends of the construction, the Chemotherapy Suite and the Oncology Ward imply a type of spatial connection which is reliant on gradual movement. One has to be removed from the Suite, with its physical association with the outer world, and re-placed to inhabit the Ward, where this association disappears. The fact becomes even more apparent in the single-bed rooms, designated as the actual places of death. These rooms have either small or no windows and the door opening is to a relative “exterior”, i.e., the corridor that makes the connection between said rooms and the nurses’ quarters. Once here, the patients have reached the end of their journey, and the architectural layout reinforces this idea.

Insofar as van Gennep’s theory is concerned, the single-bed rooms in the Oncology Ward mark a stage of reincorporation, where the patients arrive at various forms of acceptance or denial/refusal. Having negotiated the transitional process and having acknowledged their own passage from the openness of the world to the closeness of their final destinations, they may have made peace with themselves by the time they were moved to the single-bed rooms, and may have accepted that the only possible next step from there was death.

Weaving van Gennep’s theory into the findings has provided a framework within which it is possible to articulate the experiences of palliative care patients as a continuum. As such, it is relevant to a discussion about participants’ “journey” from terminal diagnosis to the end of life. This interpretation is inclusive of the idea of linearity or progression which offers a diachronic (evolving) perspective over the topic of the present article. To better articulate the argument of the current research, a synchronic perspective (observations made on discreet sites, isolated in a well-defined timeframe (20 months) was also needed. An approach of this type is provided by Miles Richardson [31].

### 3.2. Material Culture—Introducing Richardson’s Theoretical Framework

Whereas van Gennep’s theory was applied to the findings relating to the individual participants’ journeys, Richardson’s theory is accessible to observers not solely through the experiences of the participants, but is built up from the total of the observational body of data. It therefore incorporates all the participant data alongside the material set of observations.

The theoretical framework utilised by Miles Richardson [31] in his development of the concept of ‘material culture’ will be used in the discussion section to investigate the participants’ specific responses to the relational nature of place in two distinct settings: the Chemotherapy Suite and the Oncology Ward at Christchurch Hospital in New Zealand. Richardson explained that places are different from each other because they are materially, conceptually and culturally different, and people's response to these environments is informed by them and is a product of our subjective interaction with objective situations. To understand the subjective nature of human behaviour, it is necessary to regard people’s actions within the context of the particular material cultures in which they belong. Richardson uses a three-step process to analyse these interactions: “the preliminary definition supplied by the material culture of the setting; the interactions occurring within that setting; and the image emerging out of the interaction and completing the definition by restating that situation’s sense of place” ([31], p. 424).

Following the three steps outlined above, Richardson’s study provides a method for understanding the location, appearance, contents and themes of places occupied by people in general. He was particularly interested in applying Heidegger’s [35] idea of being-in-the-world to a concrete situation that invites ethnographic investigation. For this purpose, he conducted observations on two separate sites in Cartago, Costa Rica: the town’s market and its central plaza. He noted down characteristics of the two sites as well as interactions made possible by them, and analysed the results to identify significant social and cultural patterns. Richardson’s observations of these individual places are by no means limited to Costa Rica or to the social settings of Latin America. His analytical method can be expanded to capture larger and far more diverse contexts. Hence the present study’s attempt at applying his method to the context of palliative care.

Van Gennep’s concept of rites of passage implies the transition of people from the known to the unknown. Richardson’s analysis can be added to this to further develop the concept of familiarity with spatial coordinates. Familiarity with physical environments is a variable with significant consequences in the social life of individuals and groups. Unfamiliar situations cause people to pay more attention to their surroundings, while a familiar setting makes one less aware of what surrounds them. In a similar manner, when people are in private and familiar places they are less likely to be aware of their behaviour than when they are in unfamiliar environments, as their critical aptitudes are “shut down” by factors such as habit or comfort. Richardson [31] explains this change of awareness and its impact on behaviour by means of two situational concepts. He argues that people may be regarded as being off-stage in a private and familiar environment, and on-stage in an unfamiliar, often public, environment. More precisely, people perform in a more relaxed and natural manner when they are off-stage, but are more aware of their behaviour when they are on-stage and when, thus, they adjust their actions to match the perceived profile of the environment.

## 4. Discussion

### 4.1. Discussion of the Two Primary Sites

Richardson’s [31] ethnographic method of observation, which provides one of the theoretical frameworks for the present article, starts from a general consideration of two different settings to identify the qualities that make them stand out in relation to each other. The same will be done below in relation to the two sites identified throughout the research process as significant to the journey experienced by cancer patients facing end-of-life.

**Figure 2:**
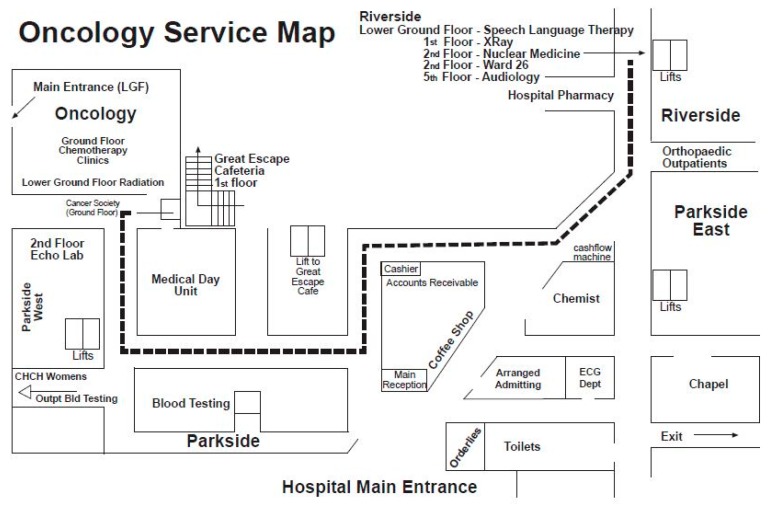
Location of the Oncology Service and the pathway to the Oncology Ward 26.

Although both the Chemotherapy Suite and the Oncology Ward are spatially separated within the hospital, they are inextricably linked (See Figure 2 below). The Chemotherapy Suite is located on the west side of the hospital and the Oncology Ward on the northeast side. As will be described below, the Suite has a direct connection with the outside world which the Ward does not have. As such, the two appear to be detached from each other. However, they are connected through their roles as signposts of stages in the palliative care continuum. This connection is spatial as well as symbolic. Patients who go through all the stages of palliative care and who, therefore, move from the Chemotherapy Suite to the Oncology Ward, experience this coming-together of the two places in terms of progression towards death. The Suite and the Ward are the two opposite ends of this continuum, and as such they also materialise the idea of spatial progression, as already explained in relation to van Gennep’s [32] transition rites, and the journey metaphor.

The Canterbury District Health Board: Oncology Services Map [36] shows the route from the Oncology Department where the Chemotherapy Suite is located and the Oncology Ward (Ward 26) in the Riverside building on the opposite side of the hospital. The source website also provides an image of the entire hospital layout (not included here).

#### 4.1.1. The Chemotherapy Suite

**Figure 3:**
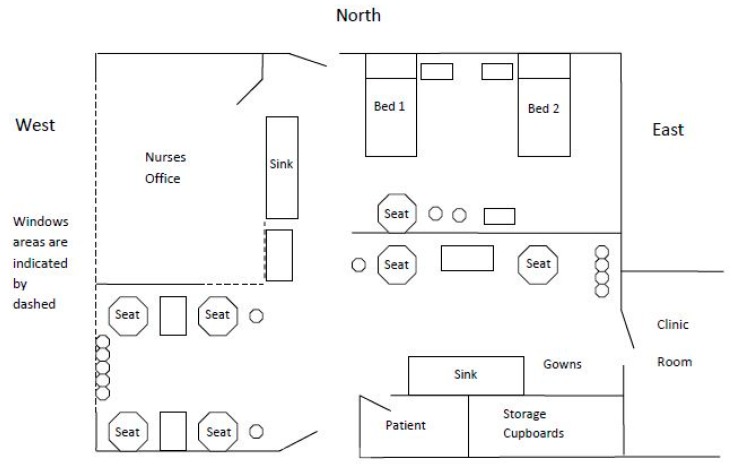
The Chemotherapy Suite. A two-dimensional view, showing patients’ and nursing staff’s location within the Suite [17].

The Chemotherapy Suite at Christchurch Hospital is a large bright room with windows that look out over the public carpark to the botanical gardens beyond. Figure 3 below provides a two-dimensional bird’s-eye view of the Chemotherapy Suite layout [17]. The walls are painted in pale pastel colours, giving a fresh, light, airy sense to the room. The nurse’s station has glass walls, which allow an open connection to the rest of the suite. This gives nurses the possibility to see people who may need them and to respond as required.

The Suite is often a very busy place, especially when all patient spaces are occupied. It is a place where many people come at the beginning of their cancer journey, when they engage in initial chemotherapy treatment. Many patients return there later on in their journey, for further treatment. New people tend to be quieter and follow the cues given to them by others about behaviour that is appropriate in that environment. They wait to be told what to do and when. After a few visits, they become enculturated to the Chemotherapy Suite and walk in the door, wave to the nurses, choose their seats and get ready for their treatment.

The Suite is a therapeutic clinical space that is open and busy during business hours and closed evenings and weekends. The nursing team and volunteers who provide cups of tea and companionship are very friendly and engaging, and become familiar with patients during treatment, often referring to them by first name. There are often gifts of appreciation in the nurses’ office, chocolates, cards and baking.

#### 4.1.2. The Oncology Ward

**Figure 4:**
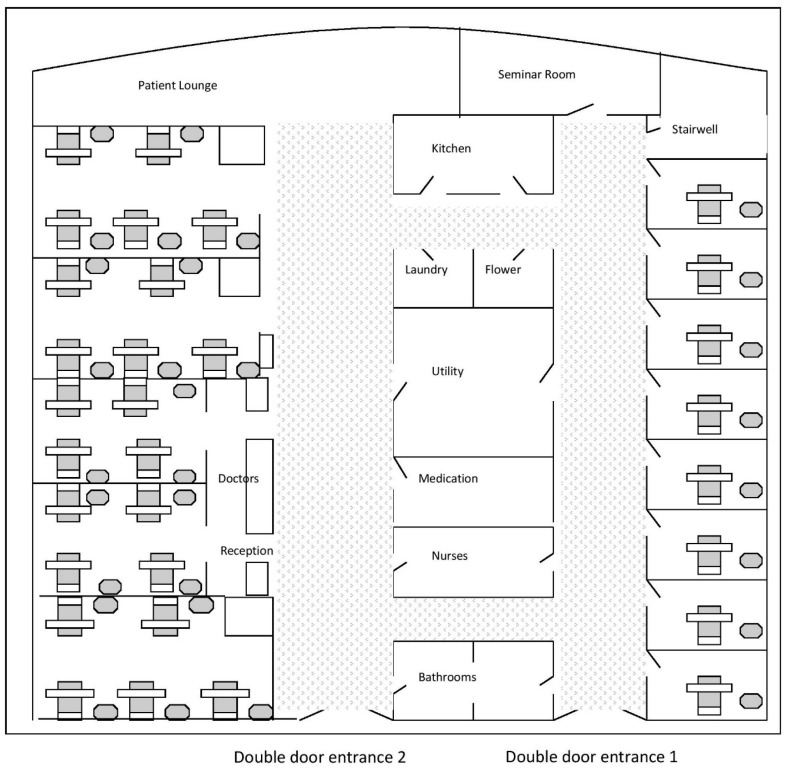
Oncology Ward. Two-dimensional view of the layout of the Oncology Ward, with its large multi-bed rooms on one side and single rooms on the other [17].

The Oncology Ward is located in the main hospital building, on the second floor. Figure 4 below provides a two-dimensional bird’s-eye view of its layout [17]. The Ward looks out over a different aspect of the botanical gardens, where there is a river and grass areas with seats for patients, who can go outside during the day. There is a significant distance between the Oncology Ward and the Chemotherapy Suite, as indicated in Figure 2. There are two entry doors to the Oncology Ward. Door one is usually closed and it leads to the single-bed rooms, where the very ill or dying are located. Door two leads to the nurse’s station and five large, four to five-bed rooms. The doors to the rooms are always open and there is often quiet chatter and nurses busy with patients or visitors in these rooms. There is a patient lounge at the end of the hallway and a patient and family kitchen is provided, as many family members spend a significant amount of time in the ward.

Like the Chemotherapy Suite, the Oncology Ward is painted in pale pastel shades, with cream curtains delineating the bed spaces. The curtains on the windows are patterned with flowers in muted tones. The main corridor (double door entrance 2), used as the primary entrance by staff and visitors, is approximately two meters wide and carpeted. It is a light, airy space. The patient lounge at the end is semi-circular, with large windows around the semi-oval back wall. On sunny days, it is a beautiful room to sit in and look out over the river. The other corridor, on the opposite side of the ward, has closed double doors (entrance 1) and is decorated in the same tones and has the same furnishings as the rest of the Ward. There are eight single-bed rooms located on this side of the Ward. At the end of the Ward, adjoining the patient lounge, is a seminar room used for staff training. This is closed to visitors, as are most of the doors to the patients’ rooms. The obstruction of access has the effect of giving this side of the Ward a darker, more hushed feel.

All the rooms are sparsely furnished with a bed, cabinet, and a couple of chairs for visitors. There are paintings of country scenes on each of the walls in the single rooms and along the large hallways, which bring a bit of colour to the pale backdrop of the Ward. There tend to be very few flowers in this ward, as many patients live a revolving-door existence and are regularly in and out of the rooms. Visitors know this and, as a result, they tend to bring fewer flowers.

The most inviting space is the patient lounge, with its recliner chairs, large windows, TV and a bookcase full of reading material and jigsaws. The large windows open about half a meter and on nice days the sounds of people out by the river or rowing down the river float up through the open windows and bring a sense of hope and joy to the room. Another area that is bright and enjoyable is the photo wall by the nurses’ station. People often stop to look at the photos of patients and nursing staff.

The general atmosphere in the Ward is very clinical and quiet, which backs up the fact that this is a place where oncology patients only go if they are very ill or suffering from health complications. They are often waiting and hoping to be discharged home. As such, it is a place of quiet rest and a place of gathering, a place where families congregate as the health of their loved ones deteriorates. The sight of families gathering outside the single rooms or in the patient lounge is very common.

The culture of each side of the Ward is quite distinct. Open doors and quiet chatter along the main corridor with the large open patient rooms, closed doors and hushed whispers or silence, prevail on the side of the ward with the single rooms. The main corridor is much busier, with doctors meeting to carry out patient rounds in the morning, the receptionist outside the nurses’ station taking phone calls and dealing with visitors. Once the doctors’ rounds are finished and the doctors have left, there may only be three or four nurses left on the ward.

#### 4.1.3. Critical Contrast of the Two Material Settings

A critical contrast of the two material settings is suggested by Richardson [31] as the next step in beginning to understand the environments under scrutiny. When it comes to distinguishing the differences that define the two settings observed in his own study (the marketplace and the plaza), Richardson articulates an objective framework for ethnographic work:

Since the ethnographer obviously wants to avoid the imposition of categories that do violence to the process he is trying to understand, the best way to handle both the “in” and “of” problem and the in-place and out-of-place distinction is to contrast a broad sample of market behaviour with that of the plaza and extract from that contrast critical interactions that illustrate the incorporation of the material setting into the emerging situations. This is attempted by making a more systematic comparison to underscore the contrast between market and plaza behaviour

([31], p. 427).

For the present study, the researchers proceeded in the same direction, attempting to single out the aspects that are characteristic to each of the two settings but absent in the other. A significant departure from Richardson’s original method must be noted here. While Richardson analysed two different places within a town, the present study regards two sites situated within the same place: Christchurch Hospital. Of course, one could argue that a hospital is a scale replica of a town. However, in Richardson’s case the two places are unrelated structurally. Their disconnection is often pointed out by the author. In the present case, however, the Suite and the Ward are connected by means of their functionality. They both address the same issue, evolution of cancer in patients who are approaching death, albeit from two different extremes. Therefore, the specificity of each site is given by their respective position relative to the evolution of the patients’ illness.

Another diversion from Richardson’s framework is that Richardson noted the behaviour of people in the market and the people in the plaza as two distinctive groups of people. The current study, however, analyses the behaviour of the same small group of participants in two different settings, often at two very different chronological stages in their illness journey.

There is a sense that people are off-stage in the Oncology Ward, and on-stage in the Chemotherapy Suite [31]. Patients in the Oncology Ward are admitted there because they are very ill or dying. They are dressed in pyjamas, dressing gowns, and slippers. Generally, visitors speak in hushed tones, whisper, or simply loiter outside the single-bed rooms in silence. The curtains around the beds are often partially or completely closed and some patients do not engage with each other, while a minority seem to make some connection. In contrast, patients in the Chemotherapy Suite are usually dressed, wearing shoes, talking, sometimes watching television, and are often quite loud and lively, and may even look quite well. Many of them look like they have come straight from work or some other activity. There is a connection to living, a sense of engagement with the world.

Richardson [31] explains that, as people encounter material locations, they develop a preliminary interpretation of appropriate “in-place” and “out-of-place” behaviour. There is meaning attached to these behaviours, which determine whether the behaviour is “in-place” rather than “out-of-place”. An example of this may be leaving a cell phone on in the Ward. If the cell phone rings, the holder would most likely be reprimanded for displaying “out-of-place” behaviour, i.e., behaviour that contravenes the norms of the Ward. Speaking in quiet and hushed tones is, by contrast, acceptable and, therefore, an instance of “in-place” behaviour. The same applies to visitors and family members who display appropriate behaviour: they receive positive reinforcement from health professionals who, thus, place the former in the ‘in-place’ category.

#### 4.1.4. Comparison of Interaction in the Two Settings

A comparison of interactions was conducted to develop a deeper systematic analysis and understanding of behaviour that occurs in the different sites. The types of interactions were determined by the level of engagement, the type of action, whether people were participants or observers, and whether they were on-stage or off-stage [31].

In the Chemotherapy Suite, the close proximity of patients adds to a sense of intense action. Nurses are busy moving between patients to set up, check on, or remove intravenous chemotherapy drips. There is often a queue of patients in the waiting room, which contributes to an additional sense of urgency. The fact that many patients are dressed in their own clothes and appear to have arrived here from other activities adds action and activity to the picture. These are people in motion, busy living, hoping for cures, and involved in the pursuit of that goal.

In contrast, the Oncology Ward can be very quiet, interspersed by ritualistic activities. Significant interactions are with doctors whose rounds tend to occur early in the morning. There is also a rest period between 1 and 3 p.m. Patients are separated by curtains within the larger rooms or are in separate rooms on the other side of the Ward. This limits interaction and contributes to the feeling of isolation. Generally, the environment is quiet, as patients tend to be quite ill when admitted here. There is also censoring of inappropriate out-of-place behaviour, such as the use of cell phones or loud conversations. These too are forms of interaction that are disapproved of in the Ward, as opposed to the Suite. On the side of the Ward with the single-bed rooms, it tends to be even quieter and there is little activity, unless family members have gathered for the death of a loved one. These families tend to speak in quiet tones, hushed whispers, and grief is often permeable.

In the Oncology Ward, where patients are caught up in out-of-the-world situations, the physical environment is broken into multiple sites, as opposed to the openness of the Chemotherapy Suite. This results in multiple sites of activity, including the corridors, the large bedrooms, the single bedrooms, the patient lounge, and the nurses’ station. Action tends to be slow and patients often spend a significant amount of time focusing on the ward’s surroundings. Although the clinical décor of the Chemotherapy Suite and the Oncology Ward are similar, the lack of interaction in the Ward results in patients’ commenting on the lack of vibrancy of the décor.

Alice has been admitted to the Oncology Ward. When I visit, she explains that she is writing a letter to the hospital board about the colour of the room and how she calls it the death ward. I said to her, well if you have to be admitted again, I will do what I can to brighten up the room.

[33]

As made apparent by the collected data, the décor of the Chemotherapy Suite tends to be the background to activity, while the décor of the Oncology Ward tends to become the patients’ focus. As far as this distinction is concerned, it is important that we draw attention to another difference between the present study and the findings of Richardson’s [31] research. While Richardson focused on interactions observed in the two settings he analysed, the current study also considers the shift of emphasis from action/interaction to lack of action and the resulting focus on the material location. Once again, this is due to the nature of the two sites analysed here, which are defined by their functionality as parts of the same continuum, and not by their functional difference, as was the case in Richardson’s accounts. As patients’ progress from the comfort and familiarity of the outside world towards the certainty of death, encountering, mid-way, the treatment stage performed in the Chemotherapy Suite, their levels of interaction are gradually reduced, as well as their ability to carry on daily routines. Their lives are no longer active but contemplative. They do less and observe more.

Richardson’s analytic method considers interactions as crucial to an ethnographic situation, if more difficult to record. “[O]ut of the ongoing process of interaction emerges a sense of the situation that is being defined” ([31], p. 431). To apply this principle to the present study, one needs to understand that both Chemotherapy Suite and Oncology Ward are places where people wait. The wait in the Suite is shorter and more interactive, while in the Ward it is often measured in days. In the Suite there is action, treatment, hope, stories being told, experiences being shared, and a sense of connection with life and living. In the Ward, by contrast, patients are waiting for operations, treatment, test results; they are waiting to get better, waiting to go home, or waiting to die. The staff-to-patient ratio is higher in the Chemotherapy Suite, which explains the higher level of action and activity. The lower ratio in the Oncology Ward may also contribute to patients’ feelings of loneliness and abandonment. Interestingly, boundary issues are more prominent in the Ward, where there is greater proximity between patients, compared to the Chemotherapy Suite, where the physical distance is much shorter. More precisely, patients do not have a choice of who they sleep next to in the Ward, while in the Chemotherapy Suite they do enjoy relative freedom with regards to choosing their proximities. Obviously, sharing an environment with other people for days is more challenging than having to do the same over a span of a few hours. Moreover, both sites are public domains and are therefore devoid of explicit privacy. While this did not appear to be an issue in the Chemotherapy Suite, it did appear to be an issue in the Oncology Ward, where there is more interaction with loved ones, who may be present there for extended periods of time. In the Suite, patients may be accompanied by friends, family members, or Cancer Society drivers, who may or may not stay for the duration of the treatment. These people are present at the invitation of the patient, who may have asked them to take them to hospital for the treatment. This comes in contrast to the Oncology Ward, where anyone can appear during visiting hours and gain access to the patient. As a result, very ill patients are often seen attempting to deal with more visitors here than they would have to manage if they were at home. This contributes to a perceived lack of privacy, when patients see their lives played out in a public domain.

#### 4.1.5. Theoretical Considerations of Interactions

Moving from a preliminary definition of the setting to a “full exposition” of the interactions within the setting is the next step in analysing interactions occurring in a place. This results in the development of an image that completes the definition. As Richardson states,

The final step in the process of incorporating the setting into the ongoing situation is the objectification of the sense of the situation upon the setting so that the setting becomes a material image of the emerging situation

([31], p. 85).

As the ethnographer completes their analysis, they develop a material image of the environment. At the beginning of analysis, the participants were referred to as “people”. However, as the analysis progressed and was located within the hospital environment, they became known as “patients”, a term that implies that they are ”of” the setting, as opposed to being “in” the setting. This distinction, with its implications of transition similar to those found in the rites-of-passage framework [32], is more noticeable in the Oncology Ward, where patients are clearly delineated as being “of” the setting because they cannot leave the Ward without permission, they are not dressed in clothes to go out, but clothes to stay in. They are expected to be on or near their beds and easy to locate. Interdictions (an order not to do something by a person with authority) permeate this place. The rites of passage performed here are concerned with the reinforcement of the idea of ending, of reaching a stage where one’s agency is taken away.

By contrast, patients can be more accurately regarded as being “in” the Chemotherapy Suite, where they inhabit the place for a limited period before re-joining the world at large. They do not belong here, and as a result, the relationships they engage in are more open and in more direct reference to the outside world. The rites performed here are of the integrative type, as hope and the promise of recovery are the central factors. Hence the focus on administrative tasks (check-ins, tests, treatments, filling of forms etc.).

Insofar as the on-stage/off-stage distinction in Richardson [31] is concerned, the Chemotherapy Suite, with its uniquely arranged reclining chairs and beds placed within close proximal distance of each other, sets the scene for greater levels of interaction, which are intense and on-stage. The Oncology Ward, with its separated bed spaces, sets the scene for less interaction, creating an environment that is serene and off-stage. The theme of illness is implicit here; this is the place where they come to wait. On one side of the Ward they wait for treatment, test results, to get better, and go home. On the other side, they wait for bad news or the inevitability of death. During one of the hospital visits to Alice, she was found in one of the large, five-bed rooms, talking about a lady who was sharing the room with her that morning, but who had a fall and had now been moved to a single room on the other side of the ward.

Alice says, I know she wasn’t good but I was just talking to her 5 min before she fell. I know people die in hospitals, but you just don’t expect to see it. I didn’t know she had died but the nurse told me when I asked where she had gone. After she fell, her family all arrived and were here for four hours. They had moved her to the side rooms, so I should have known.

[33]

Alice’s statements serve as evidence of the tension that pervades the Oncology Ward and of the degree to which patients come to inhabit the conventions of the place to the point where they identify with the process. “I should have known” indicates that Alice had become sufficiently acquainted with the rites of passage performed here (by being, in other words, “of” the Ward) to be able to make predictions.

As a general observation, it could be said that the two different environments contrasted here became places for interaction, and not simply locations. Each environment contained a particular meaning and type of interaction. Gaining an understanding of the two locations allows for the development of a statement which explains the “overall meaning of the two places” [31].

#### 4.1.6. Contrast of the Two Images

The final step of the overall analysis proposed by Richardson is a contrast of the two images. In the Chemotherapy Suite, medicalisation triumphs over death. Even when people such as the participants have been told that the cancer is incurable, they come to the chemotherapy suite holding hope in their outstretched hands. Life is viewed as a precious commodity to be extended, regardless of the cost. Extra days are carved out of time. People are actively engaged in the fight against cancer and they are aware that if they do not participate they may die. Sometimes they will succeed and sometimes they won’t.

In contrast to this image of medicalised dominance over illness, disease predominates in the Oncology Ward. The two places have almost polarised meanings, yet they are inextricably linked. Engaging in chemotherapy treatment can lead to admission to the Ward for management of side-effects, and not having chemotherapy treatment can lead to admission to the Ward for symptom management.

Although the themes of the two places may appear to be mutually exclusive, they are not. However, the existential meanings of the two environments can create a dichotomy in the minds of patients. People who engage in chemotherapy treatment or who are admitted to the Ward “have two distinct realities in which to be” [31]. Often, the fight for more time, which occurs in the Chemotherapy Suite, takes priority over the consideration of how much time is left. This can lead terminally-ill patients to neglect the need for considering death and dying.

## 5. Conclusions

With a predicted increase in the ageing population as a result of the post-WWII baby boom, conversations relating to the health system have never been timelier. To develop adequate and responsive systems of care we need to understand how places affect people and how people affect places. Despite this, no literature concerned with palliative patients’ occupation of spaces within the hospital was identified. The current study can be utilized as a foundation for further exploration of palliative patients’ experiences within the health care system, and could also make a valuable contribution to the design of health facilities. Consideration must also be given to decisions relating to the care and treatment of patients with a palliative diagnosis, as late-stage palliative treatment may result in higher rates of hospitalisation due to acute care needs. Patients develop profound knowledge of their health status and the health system, as they move from being “in” to be “of” the system. During the rite of passage from diagnosis to the end-of-life patients become increasingly sensitized to the nuances of the environments they occupy. Care about how spaces for the end-of-life are designed must be taken to adequately provide environments in which the very ill or dying feel emotionally, physically, and spiritually supported by hospital staff and their extended networks of family and friends.

## Figures and Tables

**Table 1:** Time spent with participants and their family networks- in minutes. Figures in bold along the bottom of table one indicate the total minutes spent in each location. The sub-total column indicates how much time was spent with each participant. Adding the writing up time to the sub-total provides a total data collection figure for each of the eight participants.

Participant	Phone	Home	Oncology Clinic	Chemotherapy Suite	X-ray etc.	Oncology Ward	Local Hospice	Funeral	Travel	Sub-Total	Writing up	Total
										Contact Time		Data Collection
1	57	1037	715	0	365	1530	0	120	1490	5314	4641	9955
2	85	1155	150	1350	0	1865	0	720	2220	7545	7282	14,827
3	62	481	0	0	0	60	0	0	150	753	1326	2079
4	135	3760	2520	2154	1170	2765	135	1095	5640	19,374	15,244	34,618
5	45	2419	270	1385	0	140	0	120	2480	6859	7386	14,245
6	45	890	120	0	0	1055	0	120	980	3210	2934	6144
7	320	1800	480	0	0	0	15,510	900	4220	23,230	33,294	56,524
8	50	140	0	0	0	0	360	240	205	995	1090	2085
	**799**	**11,682**	**4255**	**4889**	**1535**	**7415**	**16,005**	**3315**	**17,385**	**67,280**	**73,197**	**140,477**

**Table 2:** Time spent with participants and their family networks. Totals, in minutes, from each column in Table one (in bold) have been converted into hours, days and weeks. The sub-total column indicates the total amount of contact time in minutes (bold), then hours, 8 h days, 5 day weeks, and months. Adding the writing up time to the sub-total provides a total data collection figure. In total 14.6 months of 8 hour days, five days a week was spent with the participants.

Time	Phone	Home	Oncology Clinic	Chemotherapy Suite	X-ray etc.	Oncology Ward	Local Hospice	Funeral	Travel	Sub-Total	Writing up	Total
										Contact Time		Data Collection
**Minutes**	**799**	**11,682**	**4255**	**4889**	**1535**	**7415**	**16,005**	**3315**	**17,385**	**67,280**	**73,197**	**140,477**
hours	13.32	194.7	70.92	81.48	25.6	123.59	266.75	55.12	289.75	1121.23	1219.95	2341.18
8-h days	2	24.3	8.8	10.185	3.2	15.44	33.34	6.89	36.21	140	152.49	293
5-day weeks	0.33	4.9	1.8	2.04	0.64	3.1	6.7	1.4	7.2	28.11	30.5	58.61
Months	0.08	1.2	0.5	0.5	0.2	0.8	1.7	0.4	1.8	7	7.6	14.6
